# Differing deregulation of EGFR and downstream proteins in primary colorectal cancer and related metastatic sites may be clinically relevant

**DOI:** 10.1038/sj.bjc.6604848

**Published:** 2009-03-17

**Authors:** F Molinari, V Martin, P Saletti, S De Dosso, A Spitale, A Camponovo, A Bordoni, S Crippa, L Mazzucchelli, M Frattini

**Affiliations:** 1 Institute of Pathology, Locarno, Switzerland; 2 Oncology Institute of Southern Switzerland, Ospedale San Giovanni, Bellinzona, Switzerland; 3 Ticino Cancer Registry, Locarno, Switzerland

**Keywords:** colorectal cancer, epidermal growth factor receptor, *K-Ras*, *BRAF*, PTEN

## Abstract

Cetuximab and panitumumab efficacy in metastatic colorectal cancer (mCRC) may be influenced by *EGFR* gene status and/or deregulation of its downstream signalling proteins detected in primary tumour. However, metastasis might have different molecular patterns with respect to primary tumour, possibly affecting the prediction of EGFR-targeted therapy efficacy. We analysed primary tumour and metastasis in 38 mCRC patients. Twelve cases were cetuximab/panitumumab treated. *EGFR* gene status and protein expression were investigated through fluorescent *in situ* hybridisation and immunohistochemistry (IHC), *K-Ras*/*BRAF* mutations by sequencing and PTEN expression by IHC. We observed *EGFR* gene deregulation in 25 out of 36 primary tumours and 29 out of 36 metastases, *K-Ras* mutations in 16 out of 37 cancers and in 15 out of 37 metastases, *BRAF* mutations in 2 out of 36 cancers and 2 out of 36 metastases and PTEN loss in 8 out of 38 cancers and 12 out of 38 metastases. For the first time in literature, we show that primary colorectal cancer and paired metastasis may exhibit difference with respect to EGFR pathway deregulation mechanisms possibly implying a different response to cetuximab or panitumumab treatment. The investigation of treated patients confirms this hypothesis. We therefore suggest that the analysis of metastatic lesion should be considered in patient management as well as in designing future clinical trials aimed to investigate the effect of anti-EGFR monoclonal antibodies in the treatment of mCRC.

Epidermal growth factor receptor (EGFR) is a member of the transmembrane tyrosine kinase receptor family ErbB, involved in controlling cell growth, differentiation and proliferation by triggering both the Ras–RAF–MAP kinase pathway and the PI3K–PTEN–Akt pathway (Ciardiello and Tortora, 2008). Epidermal growth factor receptor constitutive activation leads to malignant transformation, angiogenesis and metastatic dissemination. Owing to its relevance in cancer development, EGFR represents a natural molecular target for a new class of anticancer drugs. In colorectal cancer, where EGFR is overexpressed in a consistent number of cases, cetuximab and panitumumab, two monoclonal antibodies (MoAbs) that recognise the extracellular domain of the receptor leading to its inactivation, have entered in clinical practice for the treatment of metastatic disease (Ciardiello and Tortora, 2008). Both drugs, however, are effective only in approximately 10% of metastatic colorectal cancer (mCRC) patients (Ciardiello and Tortora, 2008), underscoring therefore the need of simple tests able to predict a response to these agents.

To date, several studies demonstrated that EGFR protein expression detected by immunohistochemistry (IHC) in cancer specimens is insufficient to determine response to cetuximab therapy (reported in Chung *et al*, 2005). By contrast, *EGFR* gene copy number gain (CNG, due to either polysomy or gene amplification), evaluated by fluorescent *in situ* hybridisation (FISH), seems to be a better predictive marker for anti-EGFR MoAb sensitivity (Lièvre *et al*, 2005; Moroni *et al*, 2005; Cappuzzo *et al*, 2007; Frattini *et al*, 2007; Sartore-Bianchi *et al*, 2007; Personeni *et al*, 2008), whereas the presence of *K-Ras* mutations and/or loss of PTEN protein expression by IHC predicts resistance to these drugs (Lièvre *et al*, 2005; Moroni *et al*, 2005; Benvenuti *et al*, 2007; Cappuzzo *et al*, 2007; Di Fiore *et al*, 2007; Frattini *et al*, 2007; Khambata-Ford *et al*, 2007; Sartore-Bianchi *et al*, 2007; De Roock *et al*, 2008; Lièvre *et al*, 2008).

All these data have been obtained by analysing clinical response in mCRC patients with regard to molecular features detected in primary tumour. It is possible, however, that primary tumour and paired metastatic lesions might be different at the molecular marker expression or gene status levels and that these differences may affect the clinical significance of a predictive test. In this contest, it is noteworthy that the few previously published studies on this issue focused almost exclusively on the rather unreliable evaluation of EGFR expression by IHC (McKay *et al*, 2002; Ooi *et al*, 2004; Scartozzi *et al*, 2004; Bralet *et al*, 2005; Italiano *et al*, 2005; Bibeau *et al*, 2006; Cappuzzo *et al*, 2007). The aim of this study is to analyse molecular alterations predictive for anti-EGFR therapies response, such as *EGFR* gene status, *K-Ras* and *BRAF* mutations, and PTEN protein expression, in primary tumour and synchronous or metachronous metastasis. In patients treated with MoAbs against EGFR, the molecular and clinical data were matched.

## Patients and methods

### Patient population and treatment regimens

The analysis was conducted in 38 patients who underwent primary surgery for colorectal cancer presenting with synchronous or developing metachronous metastasis and who were identified from the database of the local cancer registry (www.ti.ch/tumori). Tissue specimens were available for both primary tumour and metastasis, and they were evaluated at the local institute of pathology (www.ti.ch/icp) after fixation in 4% neutral buffered formalin. All tumours were adenocarcinomas. Twelve patients were treated with cetuximab- or panitumumab-based regimens at the Oncology Institute of Southern Switzerland. With the exception of one patient who received cetuximab as a frontline therapy, the others had failed at least one prior chemotherapy regimen based on irinotecan. For the last patients, the MoAbs were administered in combination with irinotecan given at the same dose and schedule as previously used. Treatment was continued until progressive disease (PD) or toxicity occurred, according to the standard criteria (Therasse *et al*, 2000) or to specific trial guidelines. Patients evaluated in this study were selected based on evidence that treatment outcome could be attributable only to administration of either panitumumab or cetuximab.

### Clinical evaluation and response criteria

The response was assessed every 6 weeks with radiological examination (computerised tomodensitometry or magnetic resonance imaging). The Response Evaluation Criteria in Solid Tumors were adopted for evaluation and classified as partial response (PR), as stable disease (SD) or PD. Patients with SD or PD were defined as non-responders (NRs) (Therasse *et al*, 2000). Response to therapy was also evaluated retrospectively by independent radiologists.

### Molecular analyses

All formalin-fixed paraffin-embedded tumour blocks were reviewed for quality and tumour content, and a single representative tumour block from each case, containing at least 70% of neoplastic cells, was selected for immunohistochemical, cytogenetic and molecular analyses. Tumour macrodissection was performed in tumour blocks containing less than 70% of neoplastic cells (to reduce the presence of non-neoplastic tissues). Genomic DNA was extracted using the QIAamp Mini kit (Qiagen, Chatsworth, CA, USA) according to the manufacturer's instructions.

#### Immunohistochemistry:

Epidermal growth factor receptor protein expression was evaluated on 3 *μ*m thick tissue sections using the EGFR pharmDx assay (Dako Cytomation, Carpinteria, CA, USA) according to the manufacturer's instructions, as reported earlier (Frattini *et al*, 2007). Tissue samples were considered EGFR positive if at least 1% of malignant cells were stained for EGFR. As external controls, we used those included in the kit.

PTEN protein expression status was performed according to the literature (Saal *et al*, 2005; Frattini *et al*, 2007). PTEN protein expression was detected mainly at the cytoplasmic level, although occasional nuclear positivity was present. We considered PTEN negative those specimens showing a strong reduction or absence of immunostaining in at least 50% of cells, as compared with either the internal (normal colon mucosa) or external (normal endometrium) control.

All immunohistochemical analyses were performed by two independent observers (SC and LM) giving superimposable results. The evaluation was performed without knowledge of clinical evaluation of the results of other analyses.

#### Fluorescent *in situ* hybridisation:

*EGFR* gene status evaluation was performed on 3 *μ*m thick tissue sections that were treated using Paraffin Pretreatment kit II (Vysis, Downer's Grove, IL, USA) according to the manufacturer's instructions. Dual-colour FISH assay was performed using LSI *EGFR*/CEP7 probes (Vysis), as mentioned earlier (Frattini *et al*, 2007). The LSI *EGFR* probe is labelled in SpectrumOrange and covers an approximately 300 kb region that contains the entire *EGFR* gene at 7p12. The CEP7 probe, labelled in SpectrumGreen, hybridises to the *α*-satellite DNA located at the centromere of chromosome 7 (7p11.1–q11.1). To overcome the problem of tissue heterogeneity, we evaluated 10 different tumour areas and at least 10 representative nuclei from each area. Overall, a total of 100 cells for each patient were scored. For cases in which only a biopsy was available, we evaluated all the analysable nuclei. Cases showing two chromosome 7 (Chr7) in more than 50% of cells were classified as disomic. Tumour samples with an aberrant number of Chr7, defined as more than four in at least 50% of cells, were classified as markedly polysomic. Specimens with a ratio of more than 2 between *EGFR* gene and Chr7 centromere signals in at least 10% of cells were classified as carrying *EGFR* gene amplification.

The *EGFR* gene status evaluation was performed by two independent observers (FM and VM) giving superimposable results. The evaluation was performed without the knowledge of clinical evaluation of the results of other analyses.

#### K-Ras and BRAF mutational status:

We searched for *K-Ras* point mutations in codons 12 and 13, two hotspots that cumulatively include more than 95% of mutations in this gene, as already reported (Frattini *et al*, 2007). *BRAF* mutations were investigated in exon 15, in which more than 95% of *BRAF* point mutations occur, as reported earlier (Frattini *et al*, 2004). All samples were subjected to automated sequencing by ABI PRISM 3100 (Applied Biosystems, Foster City, CA, USA) and analysed with Chromas software (http://www.technelysium.com.au/chromas.html). Each sequence reaction was performed at least twice, starting from independent PCRs. In each case, the detected mutation was confirmed in the sequence as sense and antisense strands.

### Statistical analyses

The association between primary tumours and related metastatic sites for *EGFR* gene status, *BRAF* and *K-Ras* mutational status, and PTEN protein expression was evaluated by means of the Cohen's *κ*-test, appropriate for the assessment of the concordance between two categorical measurements of the same individual. A moderate and good agreement was defined as the coefficient was 0.41⩽k⩽0.60 and 0.61⩽k⩽0.80, respectively (Landis and Koch, 1977). All statistical tests were two sided. Significance levels were set at *P*⩽0.05. All statistical analyses were carried out using the SAS System V 9.1 Software (SAS Institute Inc., Cary, NC, USA).

## Results

### Patient characteristics

Patient characteristics are summarised in Table 1. Thirty-eight patients, 24 men (63%) and 14 women (37%), were included. The median age at the time of diagnosis was 67 years (range from 48 to 94 years). Twenty-nine patients had a colon cancer (76%, 14 patients in the right and 15 in the left/sigmoid colon) and nine carried a rectal cancer (24%). All, but one, patients were stage pT3/T4. Twelve carcinomas (32%) were classified as poorly differentiated and 26 (68%) as well or moderately differentiated.

Samples from metastatic sites (*n*=53) included 15 lymph nodes and 38 visceral metastases located in the liver (28 out of 38=74%), lung (3 out of 38=8%), omentum (3 out of 38=8%), peritoneum (2 out of 38=5%) and brain (2 out of 38=5%). In 19 patients, distant metastasis was confined to one site, whereas 19 patients had multiple metastases. Metastases were synchronous in 25 cases and metachronous in the remaining 13 patients.

Twelve patients were treated with MoAbs against EGFR and clinical follow-up data were available in all cases. Cetuximab or panitumumab was administered in combination with chemotherapy as upfront therapy in one case, as second line in four cases, as third line in four cases and as fourth line in three cases. Two patients (17%) achieved PR after cetuximab-based therapy (Table 2).

### EGFR protein expression

All tumour samples showed a positive EGFR expression as detected by IHC. Overall, the same pattern of EGFR protein expression between primary tumour and related metastasis, either at distant sites or in lymph nodes, was observed in all cases (*κ*=1, *P*<0.0001) (Table 2).

### *EGFR* gene status

**Figure 1:**
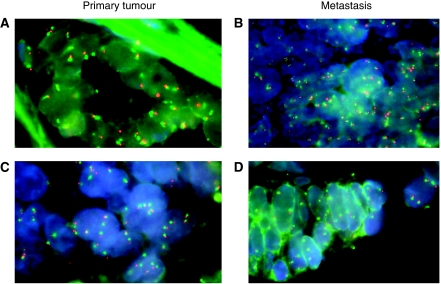
Representative examples of *EGFR* gene status differences in primary tumour and related metastasis in mCRC. Chr7 disomy in the primary tumour (**A**) and Chr7 polysomy in the paired metastasis (patient no. 6) (**B**); Chr7 polysomy in the primary tumour (**C**) and Chr7 disomy in the paired metastasis (patient no. 2) (**D**).

Two cases were excluded due to inadequate fixation of tissue sample (nos. 8 and 24, Table 2). Of the 36 remaining cases, Chr7 loss was observed in 1 (3%) primary tumour, Chr7 disomy in 10 (28%) cases, Chr7 polysomy in 17 (47%) and *EGFR* gene amplification in 8 (22%) cases (Table 2). In metastatic sites, *EGFR* gene status was classified as Chr7 loss in 1 (3%) case, Chr7 disomy in 6 (17%), Chr7 polysomy in 21 (58%) and *EGFR* gene amplification in 8 (22%) cases (Table 2). The same pattern between primary tumour and related distant metastasis was observed in 24 out of 36 (67%) patients (Table 2). Among those who exhibited differences between cancer and metastatic specimens, a trend in favour of deregulation was observed in eight cases (from Chr7 disomy to polysomy in six cases and from Chr7 polysomy to gene amplification in two cases), whereas four patients showed gene amplification or Chr7 polysomy in primary cancer and Chr7 polysomy or disomy, respectively, in related distant metastatic sites (Figure 1). If we consider the cases showing either Chr7 polysomy or *EGFR* gene amplification as a single group, we observed differences in eight patients (8 out of 36=22%), namely two cases with *EGFR* deregulation limited to primary tumour and six cases to the metastasis (*κ*=0.49, *P*=0.0002), thus revealing a moderate level of agreement between the two sites. In seven cases with a different *EGFR* gene status pattern between primary tumour and distant metastasis and for which a lymph node metastasis was available, the *EGFR* gene status in the lymph node lesion was similar to that in primary tumour in five cases and to distant metastasis in two cases (Table 2).

### *K-Ras* mutational status

One case was excluded due to the bad quality of DNA (no. 24, Table 2). Sixteen primary tumours (43%) carried a *K-Ras* point mutation, of which 13 occurred at codon 12 and 3 at codon 13. Mutations at codon 12 predominantly involved the second base, with prevalence of the GcT (GGT → GcT, Gly → Ala, G12A) and GaT (GGT → GaT, Gly → Asp, G12D) changes, in six and four cases, respectively, whereas one patient showed the 12GtT codon (GGT → GtT, Gly → Val, G12V). Only one patient carried a *K-Ras* mutation involving the first base of codon 12, leading to the 12aGT change (GGT → aGT, Gly → Ser, G12S). The mutations found at codon 13 corresponded to the classical transition G → A in the second base of the codon (GGC → GaC, Gly → Asp, G13D) (Table 2).

**Figure 2:**
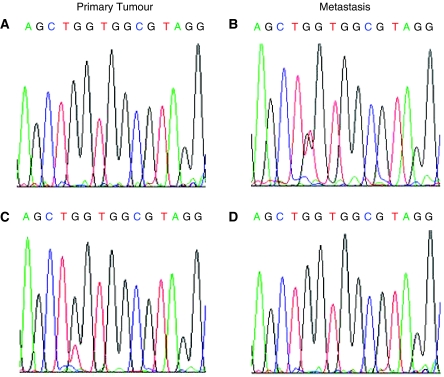
Representative examples of the *K-Ras* mutation status differences in primary tumour and related metastasis in mCRC. Wild-type *K-Ras* gene in the primary tumour (**A**) and G12C mutation in the paired metastasis (patient no. 3) (**B**). *K-Ras* G12C mutation in primary tumour (**C**) and *K-Ras* wild-type sequence in paired metastasis (patient no. 7) (**D**).

At metastatic lesions, we observed a *K-Ras* mutation in 15 patients. Overall, the same mutational pattern between primary tumour and related metastasis was observed in 34 out of 37 (92%) patients (Table 2). Among those who exhibited differences between cancer and metastatic specimens, two patients had a point mutation limited to primary tumour (one case with a 12tGT change and one with a 13GaC change) and one case to the metastasis (GGT → tGT, Gly → Cys, G12C (*κ*=0.83, *P*<0.0001)) (Figure 2).

Lymph node metastasis had a *K-Ras* mutational pattern corresponding to that observed in primary tumours.

### *BRAF* mutational status

Two cases were excluded due to the bad quality of DNA (nos. 8 and 24, Table 2). The classical *BRAF* point mutation occurring at codon 600, leading to the amino-acid change V600E, was observed in two primary tumours (2 out of 36=6%) and in the 2 (6%) related metastatic lesions (Table 2). Overall, the same mutational pattern between primary tumour and related metastasis, either at distant sites or in lymph nodes, was observed in all cases (*κ*=1, *P*<0.0001) (Table 2). In the two patients carrying a *BRAF* mutation we did not detect any point mutation in the *K-Ras* gene (Table 2).

### PTEN protein expression

**Figure 3:**
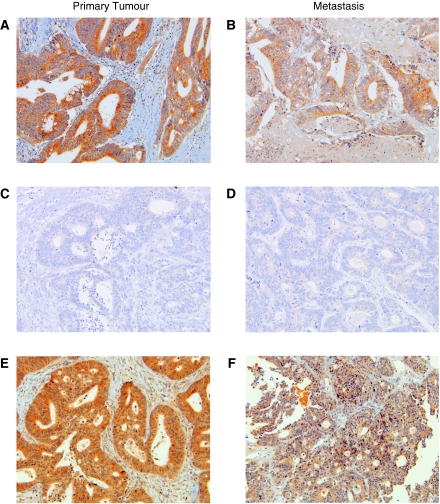
Representative examples of PTEN expression in primary tumour and related metastasis in mCRC. Equal PTEN immunohistochemical pattern between primary tumour (**A** and **C**) and paired metastasis (**B** and **D**): PTEN-positive expression (**A** and **B**) and PTEN-negative expression (**C** and **D**). Normal PTEN expression in the primary tumour (**E**) and reduction of PTEN immunodecoration in the metastatic lesion (**F**).

PTEN protein expression could be evaluated in all tissue samples by IHC. Normal PTEN expression was documented in 30 out of 38 (79%) primary tumour specimens, whereas a loss of PTEN was found in 8 (21%) cases. In distant metastatic lesions, 26 (68%) cases showed a normal PTEN expression, whereas 12 (32%) cases were classified as PTEN negative. Overall, the same PTEN protein expression pattern between primary tumour and related metastasis was observed in 34 cases (89%, Figure 3). Four cases with a normal expression of PTEN in primary tumour showed a complete loss of expression (nos. 8 and 36, Table 2) or a dramatical reduction of protein expression in related distant metastatic sites (nos. 28 and 31 (*κ*=0.73, *P*<0.0001), Table 2, Figure 3). Lymph node metastasis had a PTEN immunophenotype similar to that observed in distant metastatic specimens.

### Clinical response and relationship with molecular profile

Twelve patients were treated with MoAbs against EGFR and clinical data were collected. Seven patients showed a molecular profile in the primary tumour superimposable with that observed in visceral metastases (Table 2). Of these seven patients, two experienced a PR to the drug and were characterised by CNG in *EGFR* gene status and absence of any alteration in the EGFR downstream pathways. Five patients were NRs and were characterised by the absence of either *EGFR* CNG or *EGFR* CNG plus an alteration occurring in one member of EGFR downstream pathways (*K-Ras* or PTEN) (Table 2).

In five patients, all NRs, the molecular profile in primary tumour and related distant metastatic sites was discordant. Out of these, four patients showed differences in either *EGFR* gene status or PTEN protein expression (two cases each, nos. 27 and 29, nos. 28 and 31, respectively), but carried an additional mutation in the *K-Ras* or *BRAF* gene both in primary tumour and metastasis. The fifth patient (no. 36) showed loss of PTEN expression limited to the metastasis but with *EGFR* CNG, and the absence of any additional alteration in downstream pathways both in primary tumour and metastasis (Table 2).

## Discussion

Epidermal growth factor receptor-targeted molecular therapies have acquired high relevance in the treatment of mCRC. However, the administration of anti-EGFR MoAbs (cetuximab and panitumumab) prolongs the survival rate of only a subset of mCRC patients (Ciardiello and Tortora, 2008). The identification of clinical and/or pathological features, or of molecular alterations able to predict sensitivity or resistance to anti-EGFR therapies, is therefore urgently needed. Currently, the prerequisites for cetuximab or panitumumab administration are represented by the EGFR immunohistochemical overexpression and, limited to panitumumab, by the absence of *K-Ras* mutations, according to FDA and EMEA guidelines (www.fda.org; www.emea.europa.eu). Recent publications have clearly shown that the *EGFR* gene status and its downstream protein alterations (K-Ras and PTEN) may give additional information for a more accurate evaluation of anti-EGFR treatment response (Lièvre *et al*, 2005; Moroni *et al*, 2005; Benvenuti *et al*, 2007; Cappuzzo *et al*, 2007; Di Fiore *et al*, 2007; Frattini *et al*, 2007; Khambata-Ford *et al*, 2007; Sartore-Bianchi *et al*, 2007; De Roock *et al*, 2008; Lièvre *et al*, 2008). However, even the evaluation of these additional molecular markers is not able to fully predict EGFR-targeted drug response. This fact could be explained by three putative mechanisms. (i) The use of inappropriate methodologies for the evaluation of a specific molecular marker. For instance, it has been demonstrated that neither EGFR protein expression evaluation by IHC nor *EGFR* mRNA expression by RT–PCR represents the gold standard method for the assessment of EGFR deregulation (Atkins *et al*, 2004; Langner *et al*, 2004; Vallböhmer *et al*, 2005; Kersting *et al*, 2006), whereas recent data suggest that *EGFR* gene status represents a better predictive marker of anti-EGFR therapy sensitivity (Lièvre *et al*, 2005; Moroni *et al*, 2005; Cappuzzo *et al*, 2007; Frattini *et al*, 2007; Sartore-Bianchi *et al*, 2007). (ii) The involvement of other not yet investigated genes. In this context, *BRAF* may represent a novel predictive marker for anti-EGFR drug response (Benvenuti *et al*, 2007). (iii) the existence of different molecular patterns between primary tumour and paired metastatic lesion as predictive markers, so far, have been mostly evaluated in primary tumours disregarding alterations at metastatic sites. To shed light into this latter issue, earlier studies investigating primary tumours and paired metastases by IHC revealed different patterns of EGFR expression (McKay *et al*, 2002; Ooi *et al*, 2004; Scartozzi *et al*, 2004; Bralet *et al*, 2005; Italiano *et al*, 2005; Bibeau *et al*, 2006; Cappuzzo *et al*, 2007). Our data show that all primary tumours and metastases were positive, although they showed different staining intensities (data not shown).

The concept that primary tumour and distant metastasis may indeed show different molecular patterns is supported by the assessment of *EGFR* gene status by FISH. In our series, 12 cases (33%) showed a discordant pattern. We found eight cases with a trend in favour of *EGFR* gene deregulation from primary tumour to metastasis and four cases with the opposite trend. These data reflect those obtained by Italiano *et al* (2006) in non-small-cell lung cancers. Several recent studies have demonstrated that patients showing *EGFR* gene deregulation defined either by Chr7 polysomy or *EGFR* gene amplification (cumulatively named CNG) could benefit from cetuximab treatment (Lièvre *et al*, 2005; Moroni *et al*, 2005; Cappuzzo *et al*, 2007; Frattini *et al*, 2007; Sartore-Bianchi *et al*, 2007). Two groups of patients are therefore of particular interest: patients with Chr7 disomy in primary tumour and *EGFR* gene CNG in paired metastatic lesions, and patients with the opposite pattern. The first group should not be addressed to anti-EGFR MoAb treatments based on the sole primary tumour evaluation, but eventually, it may benefit from these targeted therapies because of the presence of *EGFR* gene CNG in metastatic sites. The opposite may be valid for the second group of patients. Our results demonstrate only a moderate correlation between *EGFR* gene status in primary tumours and in corresponding metastatic sites (*κ*=0.49, *P*=0.0002), and suggest that in patients with mCRC, *EGFR* evaluation by FISH performed only in primary tumour may not be accurate enough to select candidates for a targeted therapy. The heterogeneity of *EGFR* gene status in neoplastic tissues obtained at different sites from the same patient is confirmed by our findings in lymph node metastasis. In fact, in patients with differences between primary tumour and related distant metastasis, the *EGFR* gene status pattern observed in lymph nodes was superimposable to primary tumours in six cases and to distant metastasis in two cases. Our data are in contrast with those of Cappuzzo *et al* (2007), who found that in 21 out of 22 cases the FISH pattern of *EGFR* gene status in primary tumour and distant metastatic sites is similar. This difference may be explained by environmental factors or by FISH technique and evaluation criteria. With respect to the latter point, it should be mentioned that a general consensus for *EGFR* gene status evaluation by FISH is currently lacking (Martin *et al*, 2008).

We extended the comparison between primary tumours and metastatic sites to the analysis of molecular markers belonging to EGFR downstream cascade, which have been previously identified as potentially predictive for anti-EGFR MoAb efficacy.

The analysis of primary tumours revealed *K-Ras* mutations in 43% of cases, *BRAF* mutations in 6% of cases and PTEN loss of expression in 21% of cases, in agreement with the earlier data concerning sporadic colorectal cancers (Rajagopalan *et al*, 2002; Frattini *et al*, 2005, 2007). We found a concordant pattern between primary tumours and related distant metastatic sites in 34 out of 37 cases for *K-Ras*, in all cases for *BRAF* and in 34 out of 38 cases for PTEN. These data show a good agreement between primary tumours and related metastasis for the deregulation of EGFR downstream members, in line with results available in the literature for K-Ras and BRAF (Artale *et al*, 2008; Etienne-Grimaldi *et al*, 2008). However, it is important to underscore that the differences between the two sites may be clinically relevant for a given patient. In fact, patients without *K-Ras* or *BRAF* gene mutation, or with normal PTEN protein expression at primary tumour level, may become resistant if their metastases show alterations of these gene or proteins. By contrast, patients potentially resistant to the treatment may benefit from MoAb administration due to the absence of any alterations at metastatic level. The fact that primary tumour and related metastasis could be different in terms of gene mutations is confirmed by K-Ras analysis in non-small-cell lung cancer (Kalikaki *et al*, 2008).

The clinical data concerning patients treated with EGFR-targeted therapies are in line with these hypotheses. Indeed, one patient did not respond to MoAbs by virtue of the absence of PTEN expression limited to the metastatic lesion in a context of a molecular profile proficient to MoAb response (*EGFR* CNG and no additional alterations in EGFR downstream pathways in both primary tumour and metastasis). In the other four patients showing discordant molecular profiles between primary tumour and metastasis and for whom clinical data were available, the additional presence of either *K-Ras* or *BRAF* mutations at both sites predicted resistance to therapy.

It is important to highlight that we analysed mainly resection specimens (in 35 out of 38 primary tumours and in 23 out of 38 metastases), so that a discordant molecular pattern between primary tumour and paired metastasis due to a sample effect, although it cannot be excluded, is unlikely. Thus, the observed differences between primary tumour and metastasis may be explained by a trend in favour of gene or protein expression deregulation when the alteration is acquired by metastatic cells, whereas by a clone, present in a subgroup of primary tumour cells and characterised by an EGFR-independent metastatic phenotype, when the alteration is limited to primary tumour cells.

Finally, with respect to *K-Ras*, several additional issues are worthy to be discussed. First, we confirm that *K-Ras* and *BRAF* mutations are mutually exclusive (Rajagopalan *et al*, 2002). Second, besides the expected occurrence of G12D mutation (Frattini *et al*, 2007), we observed an unusual high frequency of G12A mutations, suggesting that this type of *K-Ras* alteration may confer a higher aggressive phenotype. Conversely, we found only one case with the G12V change, thus reinforcing the knowledge that this type of mutation may correlate with an indolent clinical course (Finkelstein *et al*, 1993; Frattini *et al*, 2007). However, as other studies showed discordant data (Andreyev *et al*, 2001), additional studies on this issue are clearly warranted.

To our knowledge, this is the first study concerning the analysis of EGFR protein expression and gene status, *K-Ras* and *BRAF* mutations, and PTEN protein expression in primary tumours as well as in lymph node and distant metastasis in the same cohort of mCRC patients. Our data, demonstrating different deregulation mechanisms of the EGFR pathways between primary tumours and related metastasis, deserve confirmation in larger and prospective studies. Nevertheless, the results may represent an important issue in the clinical practice and should be taken into account in designing future clinical trials based on anti-EGFR therapies.

## Figures and Tables

**Table 1:** Clinicopathological characteristics

**Patient characteristics (*N*=38)**	**Number of cases**	**Percentage (%)**
*Age*		
⩽60 years	9	24
>60 years	29	76
		
*Gender*		
Male	24	63
Female	14	37
		
*Tumour location*		
Proximal colon	14	37
Distal colon	15	39
Rectum	9	24
		
*Grade*		
Well/moderate	26	68
Poor	12	32
		
*TNM classification*		
T2	1	3
T3	21	55
T4	13	34
Unknown	3	8
		
*Metastasis site analysed*		
Peritoneal carcinomatosis	2	5
Liver	28	74
Lung	3	8
Omenthum	3	8
Brain	2	5
		
*Number of metastasis*		
Single	19	50
Multiple	19	50
		
*Metastatic lesions*		
Synchronous	25	66
Metachronous	13	34
		
*Primary tumour*		
Biopsy	3	8
Resection	35	92
		
*Metastatic lesion*		
Biopsy	15	39
Resection	23	61

**Table 2:** Immunohistochemical, cytogenetic and molecular data

	**EGFR IHC**			***EGFR* FISH**			***K-Ras***			***BRAF***			**PTEN IHC**			
**Case no.**	**T**	**LN**	**M**	**T**	**LN**	**M**	**T**	**LN**	**M**	**T**	**LN**	**M**	**T**	**LN**	**M**	**Clinical response**
1	+	+	+	P	A	A	WT	WT	WT	WT	WT	WT	+	+	+	−
2	+	NA	+	P	NA	D	G12A	NA	G12A	WT	NA	WT	−	NA	−	−
3	+	+	+	P	NV	P	WT	WT	G12C	WT	WT	WT	+	+	+	−
4	+	NA	+	A	NA	P	G12D	NA	G12D	WT	NA	WT	−	NA	−	−
5	+	+	+	P	P	P	G12A	G12A	G12A	WT	WT	WT	−	−	−	−
6	+	+	+	D	D	P	WT	WT	WT	WT	WT	WT	−	−	−	−
7	+	NA	+	P	NA	P	G12C	NA	WT	WT	NA	WT	−	NA	−	−
8	+	NA	+	NV	NA	NV	WT	NA	WT	NV	NA	NV	+	NA	−	−
9	+	+	+	P	P	P	G13D	G13D	G13D	WT	WT	WT	+	+	+	−
10	+	NA	+	A	NA	A	WT	NA	WT	WT	NA	WT	+	NA	+	−
11	+	+	+	A	A	P	WT	WT	WT	WT	WT	WT	+	+	+	−
12	+	NA	+	A	NA	A	WT	NA	WT	WT	NA	WT	+	NA	+	−
13	+	NA	+	A	NA	A	WT	NA	WT	WT	NA	WT	+	NA	+	−
14	+	NA	+	P	NA	P	WT	NA	WT	WT	NA	WT	+	NA	+	−
15	+	NA	+	D	NA	D	WT	NA	WT	WT	NA	WT	+	NA	+	−
16	+	NA	+	P	NA	P	G12D	NA	G12D	WT	NA	WT	+	NA	+	−
17	+	NA	+	P	NA	P	G13D	NA	WT	WT	NA	WT	+	NA	+	−
18	+	NA	+	P	NA	P	G13D	NA	G13D	WT	NA	WT	−	NA	−	−
19	+	NA	+	L	NA	L	WT	NA	WT	WT	NA	WT	+	NA	+	−
20	+	+	+	P	P	P	G12V	G12V	G12V	WT	WT	WT	+	+	+	−
21	+	NA	+	D	NA	P	WT	NA	WT	WT	NA	WT	+	NA	+	−
22	+	+	+	P	P	D	WT	WT	WT	WT	WT	WT	+	+	+	−
23	+	+	+	D	D	P	G12A	G12A	G12A	WT	WT	WT	+	+	+	−
24	+	NA	+	NV	NA	NV	NV	NA	NV	NV	NA	NV	+	NA	+	−
25	+	+	+	D	D	D	WT	WT	WT	V600E	V600E	V600E	+	+	+	NR
26	+	+	+	D	D	D	G12D	G12D	G12D	WT	WT	WT	+	+	+	−
27	+	+	+	D	P	P	G12A	G12A	G12A	WT	WT	WT	−	−	−	NR
28	+	+	+	P	NV	A	G12A	G12A	G12A	WT	WT	WT	+	−	−	NR
29	+	+	+	D	D	P	G12D	G12D	G12D	WT	WT	WT	+	+	+	NR
30	+	NA	+	A	NA	A	WT	NA	WT	WT	NA	WT	+	NA	+	PR
31	+	+	+	P	P	P	WT	WT	WT	V600E	V600E	V600E	+	−	−	NR
32	+	NA	+	D	NA	D	WT	NA	WT	WT	NA	WT	+	NA	+	NR
33	+	NA	+	A	NA	A	WT	NA	WT	WT	NA	WT	+	NA	+	PR
34	+	NA	+	P	NA	P	G12S	NA	G12S	WT	NA	WT	+	NA	+	NR
35	+	NA	+	P	NA	P	G12A	NA	G12A	WT	NA	WT	+	NA	+	NR
36	+	NA	+	P	NA	P	WT	NA	WT	WT	NA	WT	+	NA	−	NR
37	+	NA	+	D	NA	P	WT	NA	WT	WT	NA	WT	+	NA	+	−
38	+	NA	+	A	NA	A	WT	NA	WT	WT	NA	WT	−	NA	−	NR

Abbreviations: A=*EGFR* gene amplification; D=chromosome 7 disomy; L=chromosome 7 loss; LN=lymph node metastases; M=distant metastatic sites; NA=not available; NR=non-responsive; NV=not evaluable; PR=partially responsive; P=chromosome 7 polisomy; T=primary tumour; WT=wild-type; ‘+’=positive expression; ‘−’=negative expression.
